# Taxifolin for Cognitive Preservation in Patients with Mild Cognitive Impairment or Mild Dementia

**DOI:** 10.3233/JAD-221293

**Published:** 2023-05-16

**Authors:** Yorito Hattori, Satoshi Saito, Yuriko Nakaoku, Soshiro Ogata, Masashi Hattori, Mio Nakatsuji, Kunihiro Nishimura, Masafumi Ihara

**Affiliations:** a Department of Neurology, National Cerebral and Cardiovascular Center, Suita, Osaka, Japan; b Department of Preventive Medicine and Epidemiology, National Cerebral and Cardiovascular Center, Suita, Osaka, Japan; c Next Generation Business Development Department, Business Development Division, Towa Pharmaceutical Co., Ltd, Kadoma, Osaka, Japan; d Scientific Research and Business Development Department, Towa Pharmaceutical Co., Ltd, Settsu, Osaka, Japan

**Keywords:** Alzheimer’s disease, amyloid-β, antidementia agent, cognitive aging, mild cognitive impairment, taxifolin

## Abstract

**Background:**

The development of numerous disease-modifying drugs for age-related dementia has been attempted based on the amyloid-β (Aβ) hypothesis without much success. Taxifolin (TAX), a natural bioactive flavonoid, shows pleiotropic neuroprotective effects with inhibition of Aβ aggregation, production, and glycation, antiinflammatory effects, and amelioration of the waste clearance system. We hypothesized that TAX intake is associated with the suppression of cognitive deterioration.

**Objective:**

To investigate associations between TAX intake and cognitive changes.

**Methods:**

We retrospectively identified patients who orally took TAX 300 mg/day and regularly underwent Alzheimer’s Disease Assessment Scale-Cognitive Subscale 13 (ADAS-Cog) and Montreal Cognitive Assessment (MoCA) and compared the temporal changes in ADAS-Cog and MoCA between the non-treatment (pre-TAX) period (180±100 days) and following treatment (on-TAX) period (180±100 days) from June 2020 to November 2021. Since some additional patients underwent the Mini-Mental State Examination (MMSE) instead of the MoCA at the beginning of the pre-TAX period, the same comparison was performed using the MoCA total score converted from MMSE as a sensitivity analysis.

**Results:**

Sixteen patients were identified. TAX intake was associated with significantly higher interval changes in the MoCA subscale scores of visuospatial/executive function (*p* = 0.016), verbal fluency (*p* = 0.02), and the total score (*p* = 0.034), but not with ADAS-Cog (total score, *p* = 0.27). In the sensitivity analysis, 29 patients were included. TAX intake was associated with a significantly higher interval change in the total MoCA score (*p* = 0.004) but not with ADAS-Cog (*p* = 0.41).

**Conclusion:**

Our findings provide a basis for TAX as a novel strategy for maintaining brain health during aging. A prospective cohort study is required to confirm these findings.

## INTRODUCTION

Age-related or late-life dementia is characterized by neurodegeneration induced by the accumulation of amyloid plaques and neurofibrillary tangles, and by several overlapping features, including vascular risk factors (e.g., hypertension, diabetes mellitus, and obesity), cerebrovascular diseases, inflammation, and apolipoprotein E (*APOE*) genotypes. Only a small percentage of people beyond 80 years indeed have “pure Alzheimer’s disease (AD)” or “pure vascular dementia” [1].

Disease-modifying drugs for age-related dementia, including AD, remain unestablished despite clarification of the underlying mechanisms in basic research using animal models. In 2021, aducanumab, a monoclonal antibody that targets soluble (amyloid-β [Aβ] oligomers) and insoluble aggregates of Aβ proteins (fibrils and Aβ plaques), was granted conditional approval by the US Food and Drug Administration as the first disease-modifying therapy for the treatment of early AD [2]. Similar efficacy for early AD was also observed with the monoclonal anti-Aβ antibody lecanemab [3], although not for gantenerumab [4]. Most large clinical trials targeting only Aβ have failed to prove positive clinical effects, suggesting that Aβ reduction is insufficient for cognitive improvement. Thus, pleiotropic neuroprotective effects may be required to achieve cognitive improvement or prevent cognitive deterioration.

Taxifolin (TAX; 3,5,7,3,4-pentahydroxy flavanone or dihydroquercetin) is a natural bioactive flavonoid isolated from grapes, citrus fruits, onions, green tea, olive oil, wine, and several herbs [5]. TAX has attracted increased attention as a potential treatment for diabetes mellitus [6], cardiovascular diseases [7], several cancers (e.g., breast cancer) [8], coronavirus disease 2019 [9], and neurodegenerative diseases including cerebral amyloid angiopathy [10]. A safety profile of TAX has also been established [11]. Increasing lines of evidence suggest that TAX may have various positive effects, including antiinflammatory, antioxidant, anticancer, antiapoptosis, and mitochondrial protective effects [12]. Specifically, *in vitro* and *in vivo* studies of brain diseases have shown that TAX inhibits Aβ_40_ and Aβ_42_ aggregation [10, 13] and oligomer formation [10] and stimulates brain lymphangiogenesis [14]. Furthermore, we reported that TAX suppressed inflammation and oxidative tissue damage in the hippocampus and cortex, thereby alleviating the accumulation of triggering receptors expressed on myeloid cell 2 (TREM2)-expressing cells and reduced glutamate levels in the brains of Aβ-overexpressing mice [14]. Therefore, we hypothesized that the pleiotropic effects of TAX are clinically associated with the suppression of cognitive deterioration. Our study showed that oral intake of TAX (300 mg/day) may be a promising agent for preventing cognitive deterioration.

## MATERIALS AND METHODS

### Study design

**Fig. 1:**
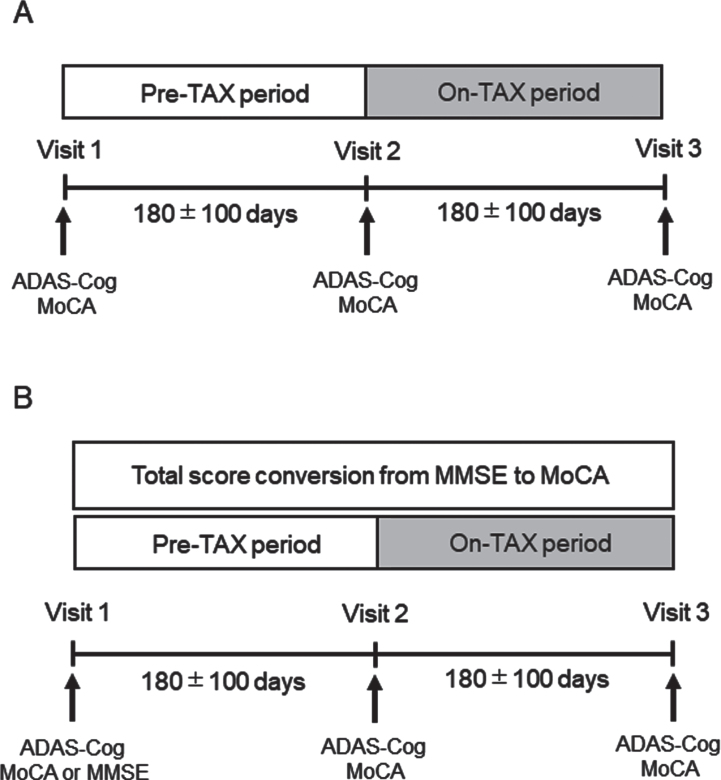
Flow chart of the study for the (A) primary and (B) sensitivity analyses.

This single-center retrospective longitudinal study was performed at the National Cerebral and Cardiovascular Center (NCVC). The study was approved by the Research Ethics Committee of the NCVC (approval number: R21047) and conducted in accordance with the Declaration of Helsinki standards. An opt-out approach was used, which meant that participants were included in the study unless they expressed their decision to be excluded. The electronic medical charts of outpatients, obtained from June 2020 to November 2021, were surveyed to identify patients who met the following criteria: 1) regularly underwent Alzheimer’s Disease Assessment Scale–Cognitive Subscale 13 (ADAS-Cog) and Montreal Cognitive Assessment (MoCA) examinations with an interval of 180±100 days as Visits 1, 2, and 3; 2) had an ADAS-Cog score of 15–37 points suggestive of mild cognitive impairment (MCI) or mild dementia [15]; 3) history of spontaneous purchase and oral intake of TAX 300 mg/day (Taxifolin Tablet, Towa Pharmaceutical, Japan) after Visit 2; and 4) provided consent for NCVC Biobank to genotype *APOE4*. The patients voluntarily purchased TAX after a clinical research coordinator provided the following information to the patients and/or their families for more than 30 min independent of the attending physicians: TAX ameliorated cognitive impairment in a study of dementia in Aβ-overexpressing mice [10], although it is unclear whether TAX was effective in patients with MCI or mild dementia. Tax tablets manufactured by Towa Pharmaceutical Co., Ltd. are commercially available. Thus, the study design consisted of two periods: the pre-TAX period between Visits 1 and 2 and the on-TAX period between Visits 2 and 3 (Fig. 1). The ADAS-Cog and MoCA were scored by three well-trained clinical psychologists (CK, AO, and MY). Hypertension, dyslipidemia, and diabetes mellitus were defined based on the administration of oral antihypertensive, antihyperlipidemic, or antidiabetic drug therapy (or insulin) prescribed by the attending physician, respectively. Antidementia drugs, such as cholinesterase inhibitors (donepezil, rivastigmine, and galantamine) and memantine and *APOE4* carrier status, which may influence cognitive function, were also surveyed in the enrolled patients. Patients whose dosage of antidementia drugs was changed during the pre-TAX or on-TAX periods were excluded. A 3-Tesla magnetic resonance imaging (MRI) scanner was used to assess background findings at Visit 1. For participants contraindicated for brain MRI because of the presence of metallic implants, such as a pacemaker device, head computed tomography was performed.

For the sensitivity analysis, we identified additional patients from the medical records from June 2020 to November 2021 who underwent the Mini-Mental State Examination (MMSE) instead of the MoCA, underwent the ADAS-Cog at Visit 1, and the MoCA and ADAS-Cog at Visits 2 and 3 with an interval of 180±100 days, spontaneously purchased and took TAX 300 mg/day after Visit 2, and provided informed consent for the NCVC Biobank to perform genotyping. The total MMSE score was converted to that of the MoCA based on the established calculation [16] when we analyzed cognitive changes between the pre-TAX and on-TAX periods (Fig. 1).

### APOE genotyping

*APOE* gene was genotyped using a fully automated gene analysis system (GTS-7000; Shimadzu Corporation, Kyoto, Japan). GTS-7000 directly detected single-nucleotide polymorphisms (SNPs) in 1μL of whole blood samples using a polymerase chain reaction [17]. We examined two SNPs, rs429358 and rs7412, that determine the *APOE* ɛ allele. The *APOE*4 was determined using rs429358-C and rs7412-C [18]. The primer sequences for rs429358 and rs7412 were 5^′^-CAAGGAGCTGCAGGCGG-3^′^ (forward), 5^′^-CAGCTCCTCGGTGCTCTG-3^′^ (reverse), and 5^′^- CGCAAGCTGCGTAAGCG-3^′^ (forward), and 5^′^-CGCGGATGGCGCTGAG-3^′^ (reverse), respectively. The probe sets were 5^′^-GGACGTGTGCGGCCG-3^′^ for rs429358-T, 5^′^- GGACGTGCGCGGCCG -3^′^ for rs429358-C, 5^′^-CTGCAGAAGCGCCTGGC-3^′^ for rs7412-C, and 5^′^-CTGCAGAAGTGCCTGGC-3^′^ for rs7412-T.

### MRI evaluation

Lacunes, white matter hyperintensities including periventricular hyperintensities (PVHs), deep and subcortical white matter hyperintensities (DSWMHs), and deep or lobar cerebral microbleeds (CMBs) were assessed according to neuroradiological findings of the small vessel diseases on brain MRI. Lacunes, white matter hyperintensities, and CMBs were identified as described elsewhere [19]. PVHs and DSWMHs were graded using a scale of 0–3 in accordance with the previously proposed Fazekas scale [20]. Regarding CMB sites, deep and lobar sites were defined according to the Microbleed Anatomical Rating Scale score [21]. The above findings were evaluated independently by two well-trained neurologists (YH and SS). The diagnosis of intracranial major artery stenosis was made based on the brain MR angiography findings according to a previously described method [22].

### Statistical analysis

All analyses were performed using SPSS software version 27 (IBM, Armonk, NY, USA) and R statistical software version 4.2.0 (R Foundation for Statistical Computing, Vienna Austria). Data on patient characteristics are summarized as median (interquartile range [IQR]) for continuous and ordinal variables and as frequencies and percentages for categorical variables. Statistical differences in the observational periods were assessed using the Mann–Whitney *U* test. Temporal cognitive changes were calculated by subtracting each subscale and total scores of the ADAS-Cog and MoCA between the pre-TAX and on-TAX periods using the Wilcoxon signed-rank sum test in the primary analysis and the paired *t*-test in the sensitivity analysis. To investigate the interactions between TAX and baseline medications related to interval changes in the total MoCA scores, we used generalized estimation equation modeling to account for patient clusters with normal distribution and robust sandwich estimates of the standard errors. All reported *p* values were two-tailed, and *p* values <0.05 were considered statistically significant.

## RESULTS

### Baseline characteristics

Sixteen patients had a history of three-times cognitive tests and TAX intake during the on-TAX period. The median (IQR) age was 77.0 (73.0–81.8) years, and six patients were men (37.5%). Five patients (31.3%) were identified as *APOE4* carriers, including one homozygous E4/E4 man and four heterozygous E3/E4 carriers (one man and three women). Seven (43.7%) patients took calcium channel blockers, six (37.5%) took angiotensin receptor blockers, two (12.5%) took β-blockers, and one (6.3%) took diuretics for hypertension. Regarding lipid-lowering drugs, seven patients (43.7%) took statins and two (12.5%) took Nieman-Pick C1-like 1 inhibitors. None of the patients took diabetic medications. The median observational periods were 150.5 (126.9–194.3) days in the pre-TAX period, and 175.0 (175.0–182.0) in the on-TAX period (*p* = 0.12). The median initial total scores at Visit 1 were 21.0 (18.3–23.8) for the MoCA and 23.7 (17.9–27.8) for the ADAS-Cog, indicating that the patients suffered from mild cognitive impairment (MCI) or mild dementia (Table 1). On brain MRI, lacunes were detected in nine (56.2%), ≥2 PVHs in four (26.7%), ≥2 DSWMHs in six (40.0%), deep CMBs in three (20.0%), lobar CMBs in seven (46.7%), and intracranial major artery stenosis in two (13.3%) patients. One patient was contraindicated for MRI (Table 2).

**Table 1:** Characteristics of the patients at Visit 1

	Primary analysis (*n* = 16)	Sensitivity analysis (*n* = 29)
Age	77.0 (73.0–81.8)	79.0 (74.0–84.0)
Male	6 (37.5)	12 (41.4)
Body weight [kg]	58.5 (51.9–68.3)	57.8 (50.1–67.8)
Hypertension	10 (62.5)	21 (62.5)
Diabetes mellitus	1 (6.3)	2 (6.9)
Dyslipidemia	11 (68.8)	17 (68.8)
Antihypertensive drug use
- Calcium channel blockers	7 (43.7)	10 (34.5)
- Angiotensin receptor blockers	6 (37.5)	9 (31.0)
- Angiotensin-converting enzyme inhibitors	0 (0)	1 (3.4)
- β-blockers	2 (12.5)	5 (17.2)
-Diuretics	1 (6.3)	3 (10.3)
Lipid-lowering drug use
- Statins	7 (43.7)	10 (34.5)
- Niemann-Pick C1-like 1 inhibitors	2 (12.5)	2 (6.9)
Antidiabetic drug use	0 (0)	0 (0)
Antidementia drug use	3 (15.8)	7 (25.0)
HbA1c [%]	5.8 (5.8–6.1)	5.9 (5.7–6.1)
TG [mg/dL]	89.0 (66.0–133.0)	103.0 (66.0–153.0)
LDL-C [mg/dL]	101.5 (69.8–115.5)	103.0 (81.0–114.0)
HDL-C [mg/dL]	58.5 (53.5–75.8)	58.5 (54.3–78.8)
*APOE4*	5 (31.3)	8 (45.5)
MoCA score at Visit 1	21.0 (18.3–23.8)	21.0 (18.3–23.8)
MMSE score at Visit 1	—	25.0 (23.0–27.0)
Converted MoCA score at Visit 1	—	20.0 (17.0–23.0)
ADAS-Cog score at Visit 1	23.7 (17.9–27.8)	24.0 (19.3–32.7)
Observational period (Pre-Tax period)	150.5 (126.9–194.3)	179.0 (129.5–196.0)
Observational period (On-Tax period)	175.0 (175.0–182.0)	175.0 (171.5–182.0)

Data are presented as the median (interquartile range) or number (%). HbA1c, hemoglobinA1c; TG, triglyceride; LDL-C, low-density lipoprotein cholesterol; HDL-C, high-density lipoprotein cholesterol; MoCA, Montreal Cognitive Assessment; MMSE, Mini-Mental State Examination; ADAS-Cog, Alzheimer’s Disease Assessment Scale–Cognitive Subscale 13.

**Table 2:** Background MRI findings of the patients

	Primary analysis (*n* = 15)	Sensitivity analysis (*n* = 27)
Lacunes	9 (56.2)	11 (40.7)
Periventricular hyperintensities
- Grade 0	1 (6.7)	3 (11.1)
- Grade 1	10 (66.7)	12 (44.4)
- Grade 2	4 (26.7)	10 (37.0)
- Grade 3	0 (0)	2 (7.4)
Deep and subcortical white matter hyperintensities
- Grade 0	0 (0)	2 (7.4)
- Grade 1	9 (56.2)	13 (48.1)
- Grade 2	6 (40.0)	11 (40.7)
- Grade 3	0 (0)	1 (3.7)
Deep cerebral microbleeds	3 (20.0)	4 (14.8)
Lobar cerebral microbleeds	7 (46.7)	12 (44.4)
Intracranial major artery stenosis	2 (13.3)	4 (14.8)

Data are presented as the number (%). MRI, magnetic resonance imaging.

### Daily intake of TAX was associated with suppression of cognitive deterioration

**Fig. 2:**
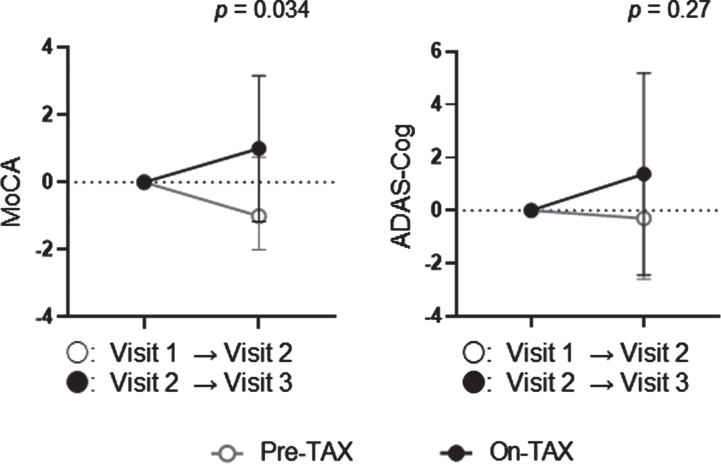
Temporal cognitive changes in total MoCA and ADAS-Cog scores. The changes in total MoCA and ADAS-Cog scores were analyzed using the Wilcoxon signed-rank sum test. Circles indicate the median scores. Error bars indicate the interquartile ranges.

**Fig. 3:**
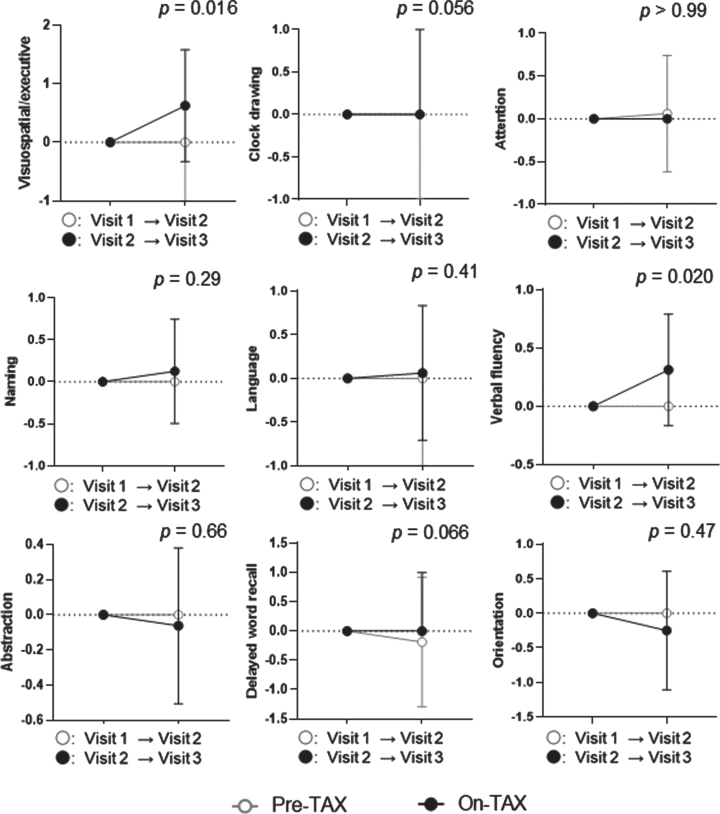
Temporal cognitive changes assessed with MoCA. The Wilcoxon signed-rank sum test showed that TAX intake was associated with significantly higher interval changes in visuospatial and executive function, and verbal fluency. Circle symbols indicate the median scores. Error bars indicate the interquartile range.

**Fig. 4:**
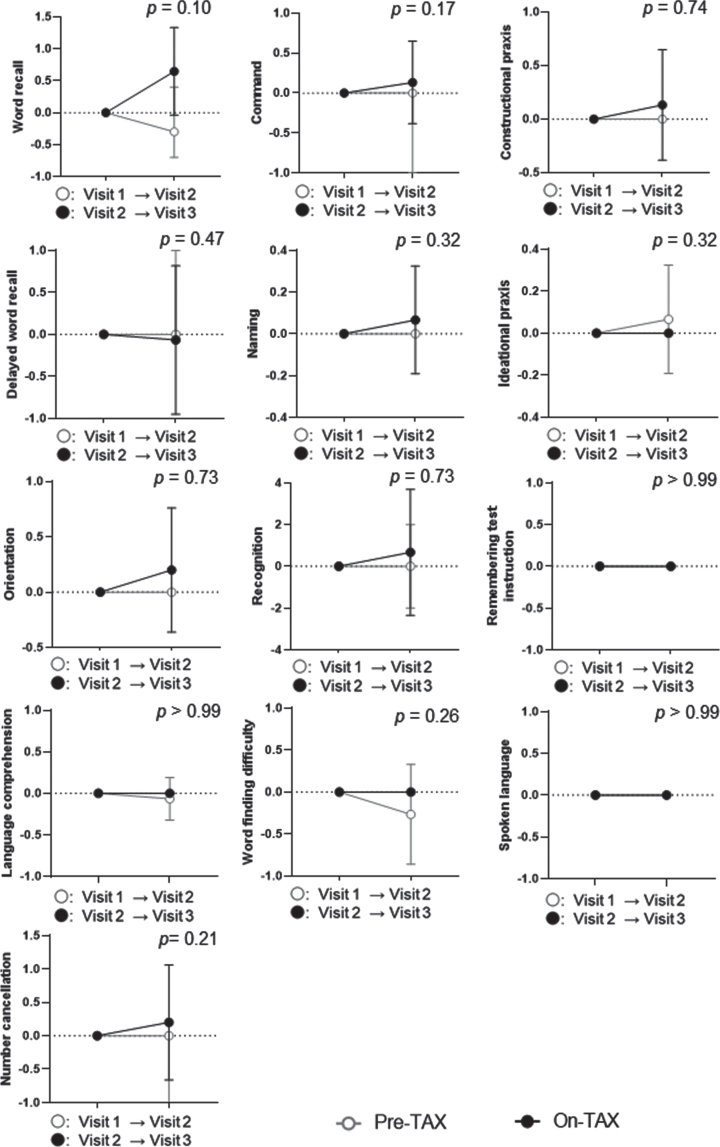
Temporal cognitive changes assessed with ADAS-Cog. No significant differences were observed between the pre- and on-TAX periods. Circle symbols indicate the median scores. Error bars indicate the interquartile range.

We evaluated the MoCA and ADAS-Cog scores to investigate cognitive changes related to TAX treatment. We observed a significantly higher interval change in total MoCA scores (pre-TAX, –1.00 [IQR, –2.00–0.75] versus on-TAX, 0.00 [0.00–2.00]; *p* = 0.034), whereas that for ADAS-Cog scores did not differ between the pre-TAX and on-TAX periods (–0.30 [–2.60–1.60] versus 0.40 [–1.70–5.00], *p* = 0.27) (Fig. 2). In exploring the changes in each cognitive domain of the MoCA, we also detected significantly higher interval changes in visuospatial/executive function (0.00 [–1.00–0.00] versus 0.00 [0.00–1.75]; *p* = 0.016) and verbal fluency (0.00 [0.00–0.00] versus 0.00 [0.00–1.00]; *p* = 0.02) (Fig. 3). Moreover, the clock drawing (0.00 [–1.00–0.00] versus 0.00 [0.00–1.00], *p* = 0.056) and delayed word recall (0.00 [–1.00–0.00] versus 0.00 [0.00–1.00], *p* = 0.066) tests also tended to show a higher interval change during the on-TAX period (Fig. 3). In contrast, the cognitive domains in the ADAS-Cog showed no significant differences (Fig. 4). The five *APOE4* carriers also showed a tendency that an interval change in the total MoCA score was higher during the on-TAX period (–1.00 [–2.00–0.50] versus 0.00 [–1.00–2.00], *p* = 0.22); however, that of ADAS-Cog did not change (–1.03 [–2.80–2.64] versus 0.40 [–0.84–5.80], *p* = 0.23). We also examined interactions between TAX and baseline medications such as antihypertensive or lipid-lowering drugs in relation to interval changes in total MoCA scores. A significant interaction was observed only in the patients taking diuretics (b = –3.33, *p* < 0.001), suggesting that the effects of TAX on the interval changes in total MoCA scores were negatively influenced by diuretics.

### Sensitivity analysis

**Fig. 5:**
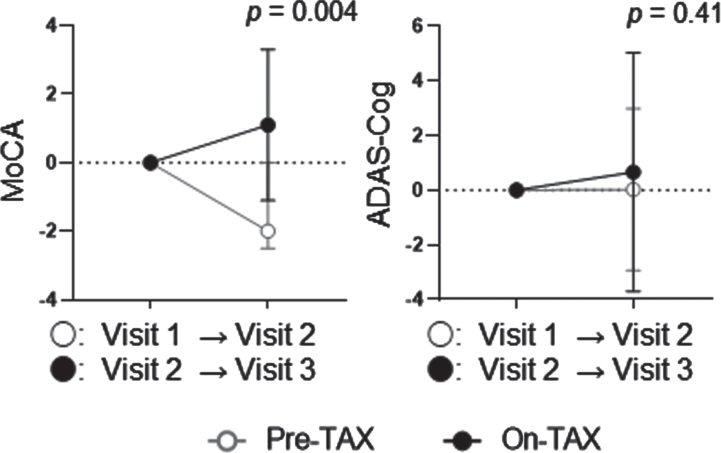
Temporal cognitive changes examined with MoCA and ADAS-Cog in the sensitivity analysis. TAX intake was associated with a significantly higher interval change in the total MoCA score. In contrast, the ADAS-Cog did not show such a change. Circle symbols indicate the mean scores. Error bars indicate standard deviation.

To confirm the effects of the daily oral intake of TAX, we conducted a sensitivity analysis that included the 16 patients mentioned above and an additional 13 patients whose MMSE scores were converted to MoCA, resulting in a total of 29 patients (12 men). The median age was 79.0 (74.0–84.0) years, and eight *APOE4* (one E4/E4 and seven E3/E4) carriers were identified. Ten (34.5%) patients took calcium channel blockers, nine (31.0%) took angiotensin receptor blockers, one (3.4%) took angiotensin-converting enzyme inhibitors, five (17.2%) took β-blockers, and three (10.3%) took diuretics for hypertension. Regarding lipid-lowering drugs, 10 (34.5%) patients took statins, and 2 (6.9%) took Nieman-Pick C1-like 1 inhibitors. None of the patients took diabetic medications. The median observational periods were 179.0 (129.5–196.0) days in the pre-TAX period, and 175.0 (171.5–182.0) in the on-TAX period (*p* = 0.94). The median initial total MoCA was 20.0 (17.0–23.0), and ADAS-Cog was 24.0 (19.3–32.7), also ranging from MCI to mild dementia at Visit 1 (Table 1). On brain MRI, lacunes were detected in 11 (40.7%), ≥2 PVHs in 12 (44.4%), ≥2 DSWMHs in 12 (44.4%), deep CMBs in four (14.8%), lobar CMBs in 12 (44.4%), and intracranial major artery stenosis in four (14.8%) patients. Two patients were contraindicated for MRI (Table 2). TAX intake was associated with a significantly higher interval change of MoCA (–1.41±2.93 versus 1.14±2.30, *p* = 0.004), but not with that of ADAS-Cog (0.032±2.96 versus 0.94±4.38, *p* = 0.41) (Fig. 5). In *APOE4* carriers, TAX intake had a tendency of association with a higher interval change of MoCA (–1.00±1.51 versus 0.50±1.14, *p* = 0.12) but not with that of ADAS-Cog (0.82±2.58 versus 2.18±5.14, *p* = 0.53). We also examined interactions between TAX and baseline medications such as antihypertensive or lipid-lowering drugs related to interval changes in total MoCA scores. A significant interaction was observed in the patients taking calcium channel blockers (b = 3.03, *p* = 0.036) and in the patients taking β-blockers (b = –3.28, *p* = 0.045), suggesting that the effects of TAX on the interval changes in total MoCA scores was influenced by calcium channel blockers or β-blockers.

## DISCUSSION

We investigated whether the oral intake of TAX was associated with the suppression of cognitive deterioration. This retrospective study showed that TAX intake was associated with significant preservation of cognitive function assessed with MoCA, especially visuospatial cognition, executive function, and verbal fluency in patients with MCI or mild dementia.

TAX, a plant-derived bioactive compound, reportedly plays a pivotal role in the inhibition and alleviation of various human ailments such as cancer, chronic hepatitis C, and cardiovascular and neurodegenerative diseases [23, 24]. Through several plausible mechanisms, TAX potentially serves as a novel disease-modifying drug in patients with neurodegenerative disorders. TAX reportedly pleiotropically targets Aβ aggregation, production, and glycation; neuroinflammation; and brain lymphangiogenic effects, thereby counteracting age-related dementia. A few *in vitro* studies have shown that TAX inhibits Aβ_40_ and Aβ_42_ fibril formation [10, 13, 25], seemingly owing to its chemical structural properties. The o-quinone structure in the B-ring of TAX binds to Aβ_42_ via lysine (Lys)^16^ and Lys^28^ residues via covalent binding. The TAX-Aβ_42_ complex suppresses Aβ_42_ fibril formation as Lys^16^ and Lys^28^ are involved in the formation of Aβ_42_ β-sheets [14, 25]. However, only a small amount of TAX passes through the blood-brain barrier when taken orally. Aβ disassembly by TAX treatment has been confirmed in *in vivo* studies. Previously, we administered TAX to vasculotropic Aβ–overexpressing mice [10]. The filter trap assay results suggested that TAX prevents the formation of toxic Aβ oligomers from monomers [10].

With regard to Aβ production, TAX inhibited Aβ production by suppressing the ApoE-extracellular signal-regulated kinase 1/2-amyloid precursor protein axis in cerebral amyloid angiopathy model mice [14], and activated Janus kinase 2/signal transducer and activator of transcription 3, and nuclear factor kappa B signaling via sirtuin 1 activation in an *in vitro* study, leading to suppressed β-secretase 1 expression [26]. The inhibitory effects of TAX on AD may be mediated by its antiglycation effect on advanced glycation end products (AGEs) since Aβ is highly glycated in patients with AD compared with that in age-matched controls [27]. Glycated Aβ shows greater neurotoxicity than authentic Aβ because glycated Aβ_42_ is more likely to increase neuronal apoptosis than non-glycated Aβ_42_ [28], while inhibition of Aβ-AGE formation by subcutaneous infusion of aminoguanidine for 3 months significantly rescued the early cognitive deficit in Tg2576 mice [28]. AGEs are stable end products formed by the reaction of proteins with the dicarbonyl compound methylglyoxal, and the interaction of methylglyoxal with arginine and Lys residues of Aβ_42_ leads to the formation of Aβ-AGEs [29]. TAX targets the Lys^16^ and Lys^28^ residues of Aβ, as indicated above, in a competitive manner, thus, glycated Aβ might be an effective therapeutic target for TAX. Therefore, TAX may play an important role in suppressing the toxicity of glycated Aβ.

Other effects of TAX may be mediated by its antiinflammatory properties. TREM2, a transmembrane protein expressed exclusively on microglia in the brain, may be implicated in the pathology of neuroinflammation and concomitant neurodegeneration such as AD and tauopathy, as shown in some *in vivo* studies [30, 31]. TAX suppresses inflammation, oxidative tissue damage, and apoptosis, thereby alleviating the accumulation of TREM2-expressing cells [14].

Finally, TAX may stimulate brain lymphangiogenic activity: we previously reported that TAX elevates the expression levels of the lymphangiogenic factors, lymphatic vessel endothelial hyaluronic acid receptor 1 (LYVE-1), and vascular endothelial growth factor-D, potentially facilitating intramural periarterial drainage (IPAD) system, which is responsible for waste clearance from the brain [14]. Furthermore, increased LYVE-1 levels might activate the meningeal lymphatic system, a putative drainage route for cerebrospinal fluid to peripheral blood, in addition to regulating the glymphatic system [32]. According to our previous study of cerebral amyloid angiopathy [10], the area (%) of Aβ_1 - 40_ accumulation is reduced in the hippocampus and blood Aβ_1 - 40_ concentrations are elevated after administration of TAX in vasculotropic Aβ-overexpressing mice. Considering these pleiotropic properties, TAX could contribute to the clearance of toxic oligomeric or glycated Aβ drainage along the IPAD pathway, in addition to inhibiting oligomer formation and glycation of Aβ. Thus, TAX may serve as a neuroprotective drug against the detrimental Aβ, neuroinflammation, and impaired IPAD systems observed in patients with dementia.

Our study showed that TAX intake may improve the MoCA subscale scores for visuospatial/executive function and verbal fluency, although not memory. The Finnish Geriatric Intervention Study to Prevent Cognitive Impairment and Disability study also showed that multidomain interventions (i.e., diet, exercise, cognitive training, and vascular risk monitoring) improves or maintains only executive function and processing speed, although not memory [33]. The ADAS-Cog mainly focuses on memory domains such as immediate word recall, delayed word recall, orientation, and word recognition [34]. The memory domain of the ADAS-Cog has a total score of 40, comprising nearly half of the overall ADAS-Cog score of 85. This may explain why TAX intake was significantly associated with MoCA but not ADAS-Cog score improvement. We considered the possible mechanisms by which TAX was associated with higher interval changes in visuospatial/executive function, verbal fluency, and total MoCA scores as follows: 1) prevention of toxic Aβ oligomer formation in the vascular walls; 2) promotion of vessel-mediated Aβ removal from the brain resulting from improved cerebrovascular reactivity; 3) reduced Aβ production through inhibition of extracellular *APOE*-extracellular signal-regulated kinase 1/2–amyloid precursor protein axis; and 4) TREM2 inhibition.

Calcium channel blockers were significantly associated with TAX in the positive interval changes of the total MoCA scores. Calcium channel blockers may be an interesting class of therapeutics as they may improve cerebrovascular perfusion to promote Aβ drainage and ameliorate Aβ-mediated increases of the intracellular calcium load by binding to presynaptic (P/Q type) or postsynaptic (L type) calcium channels [35]. Thus, calcium channel blockers may have beneficial effects for preventing dementia if coadministered with pleiotropic TAX. Conversely, coadministration of β-blockers or diuretics with TAX may not be beneficial.

Our retrospective study had several limitations. First, we compared temporal cognitive changes between the pre-TAX and subsequent on-TAX periods in the same subjects. In the pre-TAX period, the subjects received regular health advice only, but not placebo or control drugs; therefore, the observed positive effect of TAX on cognitive preservation may have resulted from a placebo effect. However, score improvement was observed in a few MoCA subscale and total scores, although not in ADAS-Cog scores, suggesting that the effects of TAX intake are cognitive domain-specific but not solely explained by a general placebo effect on cognition. Second, the sample size of this study was small; thus, comprehensive testing with larger sample size is required. Third, a clinical research coordinator explained the published information regarding the amelioration of cognitive impairment by TAX from a study of cerebral amyloid angiopathy in vasculotropic Aβ-overexpressing mice [10] to the patients before they decided to purchase TAX. A randomized controlled trial using placebo is required in the future. Despite these limitations, our results revealed that cognitive function could be maintained or even improved with oral TAX intake in patients whose cognitive function ranges from MCI to mild dementia.

### Conclusion

Cognitive function can be maintained with oral intake of TAX, which has pleiotropic neuroprotective effects in patients with MCI or mild dementia. Since disease-modifying therapies are still lacking for dementia, including AD, the development of preventive therapy with multiple targets is eagerly awaited. Our results highlight the need for a comprehensive prospective cohort study to analyze the effects of TAX on the preservation of cognitive function in patients with early-stage cognitive impairment.

## Data Availability

The data that support the findings of this study are available from the corresponding author upon reasonable request.
